# Network biomarkers, interaction networks and dynamical network biomarkers in respiratory diseases

**DOI:** 10.1186/2001-1326-3-16

**Published:** 2014-06-24

**Authors:** Xiaodan Wu, Luonan Chen, Xiangdong Wang

**Affiliations:** 1 Department of Respiratory Medicine, Zhongshan Hospital, Fudan University, Shanghai, China; 2 Shanghai Respiratory Research Institute, Shanghai, China; 3 Key Laboratory of Systems Biology, SIBS-Novo Nordisk PreDiabetes Center, Shanghai Institutes for Biological Sciences, Chinese Academy of Sciences, Shanghai, China; 4 Biomedical Research Center, Zhongshan Hospital, Fudan University, Shanghai 200032, China

**Keywords:** Network biomarkers, Dynamic network biomarkers, Lung cancer, Diagnosis, Prognosis

## Abstract

Identification and validation of interaction networks and network biomarkers have become more critical and important in the development of disease-specific biomarkers, which are functionally changed during disease development, progression or treatment. The present review headlined the definition, significance, research and potential application for network biomarkers, interaction networks and dynamical network biomarkers (DNB). Disease-specific interaction networks, network biomarkers, or DNB have great significance in the understanding of molecular pathogenesis, risk assessment, disease classification and monitoring, or evaluations of therapeutic responses and toxicities. Protein-based DNB will provide more information to define the differences between the normal and pre-disease stages, which might point to early diagnosis for patients. Clinical bioinformatics should be a key approach to the identification and validation of disease-specific biomarkers.

## Introduction

The respiratory disease is a complex process from self-limiting to life-threatening entities such as from the chronic obstructive pulmonary disease (COPD) to respiratory failure, pulmonary embolism and lung cancer. For example, COPD will be one of the top five chronic diseases in terms of global mortality and morbidity by 2030 [1-3]. One of the major challenges in the respiratory medicine is the lack of disease-specific biomarkers for disease diagnosis, illness monitoring, therapy evaluation, and prognosis prediction. The biomarker should be a measurable indicator of normal biologic processes, pathogenic processes, or therapeutic responses, for the risk assessment, early diagnosis, and predicting and monitoring responses to therapies and toxicities [4,5]. Disease-specific biomarkers are also expected to demonstrate the disease-associated specificity, sensitivity, traceability, stability, repeatability and reliability [6]. For example, somatic mutations in the tyrosine kinase domain of the epidermal growth factor receptor was shown to be a predictive marker for a greater efficacy of gefitinib in patients with non small cell lung cancer [7,8]. However, only a few have been found to be useful clinically, although numbers of discovered and identified biomarkers are generated from preclinical research.

The development and progression of the disease may be caused from the interplay of a group of correlated molecules or a network, rather than from the malfunction of the individual gene, protein, or cell [9]. It is believed that dynamic alternations of complex interaction networks and molecular sub-networks can represent and influence responses of cells or organs to real-time changed microenvironment [10-12]. Thus, identification and validation of interaction networks and network biomarkers, especially at the protein level, become critical to develop disease-specific biomarkers for monitoring disease occurrence, progression or treatment efficacy [13-15]. The present review headlights network biomarkers, interaction networks, dynamical network biomarkers, with special focus on respiratory diseases, with an emphasis to integrate bioinformatics-based screening of biomarkers, network biomarker, dynamic network biomarkers with clinical informatics and phenotypes and establish a systems biomedicine-evidenced disease-specific dynamic network biomarkers

## Network biomarkers

Gene or protein expression data and other high-dimensional profile data with over thousands of measurements in each sample can be generated from transcriptome, proteome and metabolome studies. The mining and analysis of such high-throughput data have led the current omics-based research from studying individual components to understanding functional modules or networks for biomolecular systems [16,17]. It requires multi-dimensional views and the integration of molecular interaction networks in the analysis of high-throughput data to define the variation of disease severity and progression, drug sensitivity and resistance, cell growth and differentiation, and pathogenesis elaboration. A new concept of Systems Clinical Medicine was introduced to integrate systems biology, clinical science, omics-based technology, bioinformatics and computational science to improve diagnosis, therapies and prognosis of diseases [18]. Proteomics-based bioinformatics is a critical part of the systems clinical medicine and the core approach to carry out the investigation for pathogenesis, to explore the potential of clinical applications and to improve the outcomes of patients with certain diseases.

The concept of network biomarkers was proposed as a new type of biomarkers, including a set of biomarkers and their interactions [19]. A set of high-confident biomarkers from cardiovascular-related network were identified as candidate network biomarkers and used to classify two groups of patients more accurately than current single ones without consideration of biological molecular interaction [19]. Some molecular interactions in such network could be activated under specific conditions, indicating the dysfunctional process underlying the corresponding disease phenotypes and making the detection of network biomarkers possible. Microarray analysis and human protein-protein interaction (PPI) network were combined to identify more reproducible sub-network markers than individual markers in breast cancer and achieved higher accuracy in interpretation of molecular mechanisms and classification of metastatic versus non-metastatic tumors [20].

Network biomarkers have been widely studied for early diagnosis, prognosis prediction and efficacy prediction for cancer. For example, gene expression profiling was combined with functional genomic and proteomic data from various species to generate a network containing 118 genes linked by 866 potential functional associations for breast cancer [21]. One component within the network, HMMR gene, which encodes a centrosome subunit, was discovered to be associated with the breast cancer-associated gene BRCA1 and with a higher risk of breast cancer. This network-based strategy may be used to discover additional network biomarkers for early diagnosis.

## Interaction networks

Interaction networks include gene regulatory network (GRN), PPI network, RNA network, signaling pathway network, and metabolic network. Interaction networks can provide models of cellular networks based on the integration of a large and heterogeneous dataset, e.g., from proteomics and high-throughput functional genomics studies [22]. For example, GRNs could be drawn from microarray data consisting of 62 primary tumors and 41 normal prostate tissues to explore the significant GRNs correlated with disease, severity and stage in the prostate cancer [23]. Notch1 signaling pathway was found to directly activate a feed-forward-loop transcriptional network and induce c-MYC gene expression to promot the growth of human T cell lymphoblastic leukemia cells, using an integrative systems biology approach which integrated gene expression array and ChIP-on-chip data [24]. A strategy of metabonomics based on rapid resolution liquid chromatography/tandem mass spectrometry, multivariate statistics and metabolic correlation networks was implemented to find biologically significant metabolite biomarkers in breast cancer [25]. A total of 12 metabolites were identified as potential biomarkers including amino acids, organic acids, and nucleosides. Statistical epistasis networks were inferred to characterize the global space of pairwise interactions among approximately 1500 Single Nucleotide Polymorphisms (SNPs) spanning nearly 500 cancer susceptibility genes in a large population-based study of bladder cancer [26]. The network was found to have a largest connected component of 39 SNPs that was absent in any other permuted-data networks. The observations suggested that the particular statistical epistasis network captured important features of the genetic architecture of bladder cancer that have not been described previously.

## Dynamic network biomarkers

**Figure 1:**
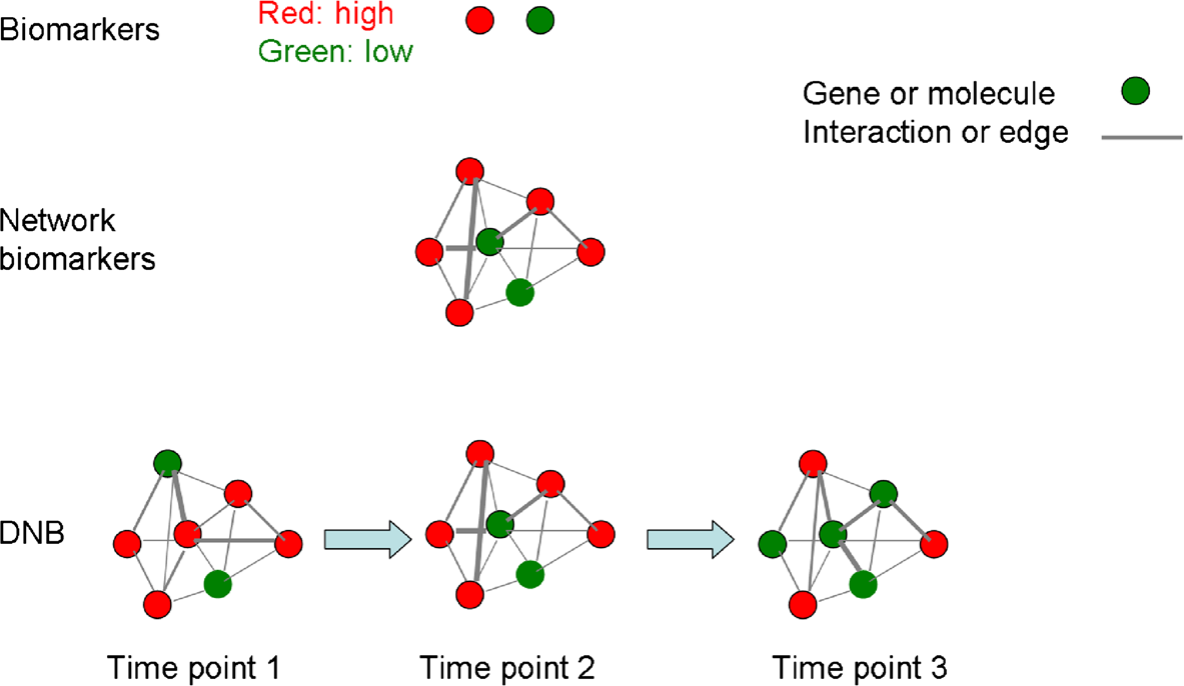
**Biomarkers, network biomarkers and dynamical network biomarkers.** Biomarkers provide one dimensional information, while network biomarkers provide two dimensional information by adding interactions. DNBs provide a three dimensional image of biomarker-biomarker interactions by showing time-dependent stronger or weaker interactions among biomarkers in the network. DNB: Dynamical network biomarkers.

Dynamical network biomarkers (DNB) show time-dependent alterations of network biomarkers monitored and evaluated at different stages and time points during the development of diseases [12]. It is more than new nomenclature, although every molecular biomarker is embedded in a molecular network and this network will show dynamic properties over time. The common objective of developing biomarkers, network biomarkers and DNB is to discover disease-specific biomarker or a panel of biomarkers for monitoring disease occurrence, progression or treatment. The study on network biomarkers emphasizes the interaction among molecules, while DNB stresses dynamical alterations of biomarkers to provide more precise and intact view for biomarkers mining. The expression levels of biomarkers can provide one dimensional information, network biomarkers provide two dimensional information by adding interactions of biomarkers, and DNB provide a three dimensional image of biomarker-biomarker interactions by demonstrating the location and time of altered biomarkers, and time-dependent stronger or weaker interactions among biomarkers in the network [12]. The integration of bioinformatics-based dynamic network biomarkers with clinical informatics and phenotypes is expected to provide a four-dimensional image of systems biomedicine-evidenced disease-specific dynamic network biomarkers. The differences among molecular biomarkers, network biomarker and DNB are shown in Figure 1.

**Figure 2:**
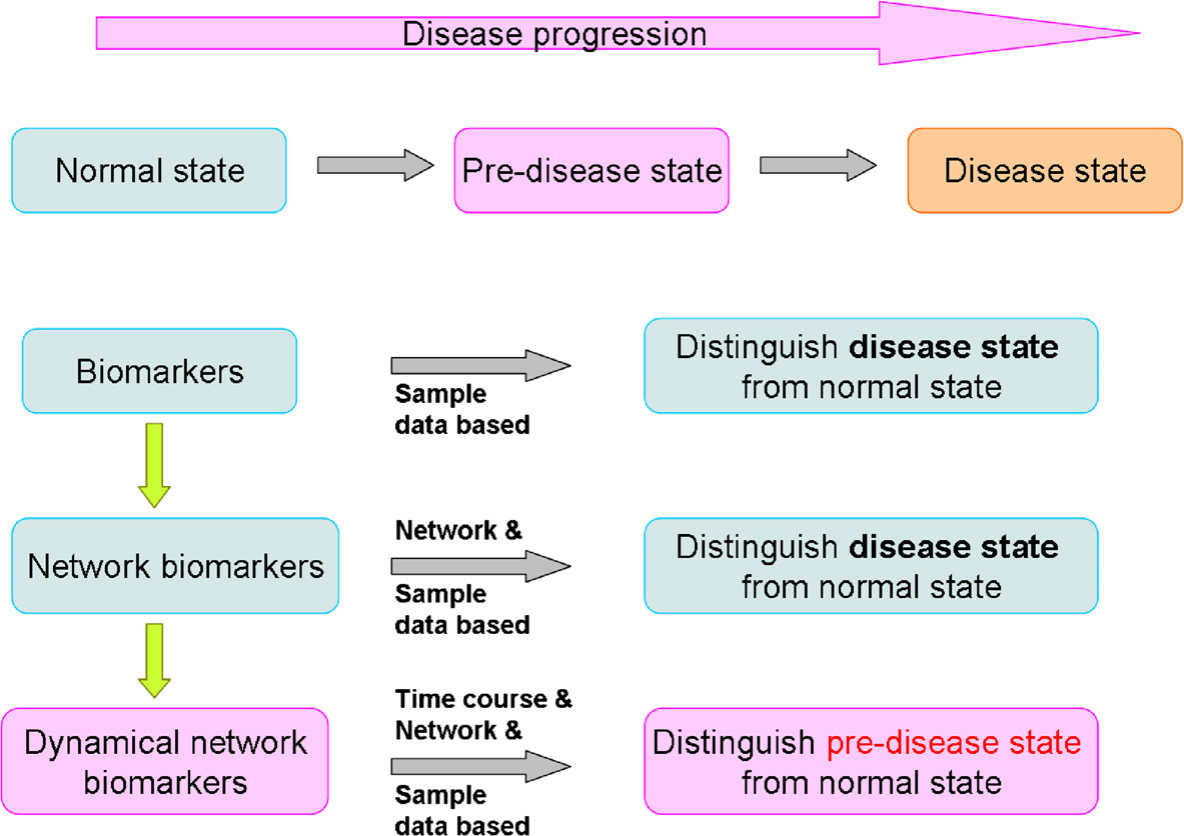
**Disease states and biomarkers.** There are three stages during disease progression, i.e., a normal state, a pre-disease state and a disease state. A normal state is a relatively healthy stage including the chronic inflammation period or the stable period during which the disease is under control, whereas a pre-disease state is the limit of the normal state just before the critical transition into the disease state. And there are three types of biomarkers, i.e., traditional molecular biomarkers, network biomarkers, and newly developed dynamical network biomarkers (DNB). Both molecular and network biomarkers are static measurements on the disease and indicators on the disease state, whereas DNB are dynamical measurements on the pre-disease, thus providing the early-warning signals for the pre-disease state.

We have emphasized to detect early-warning signals of the “pre-disease state” to prevent the critical transition from normal to disease state and achieve the early diagnosis and intervention for a complex disease. Biomarkers, network biomarkers, and DNB have respective functions in distinguishing normal, pre-disease, and disease stages during disease progression, as illustrated in Figure 2. Biomarkers or network biomarkers are mainly applied to distinguish the normal and disease situation, while DNB were proposed to be able to identify a pre-disease state even with small amounts of samples, provided that high throughput data were available for each sample [27,28]. Tissue-specific early warning signals were identified using DNB theory during type 2 diabetes mellitus (T2DM) development and progression [29]. Other than two different critical states characterized as responses to insulin resistance and serious inflammation, a new T2DM-associated function, i.e. steroid hormone biosynthesis, was also discovered. DNB could signal the emergence of the critical transitions for early diagnosis of diseases, and provide the causal network of the transitions for revealing molecular mechanisms of disease initiation and progression at a network level.

DNB have advantages over network biomarkers [16], e.g. make early diagnosis possible, develop biomarkers from a small number of samples based on a model-free method, or be relatively easy for clinical application. In addition, the concept of DNB will be generally applicable for detecting early-warning signals of critical transitions in any other biological process, e.g. cell differentiation processes, aging processes, and phase changes of cell cycle. Dynamic networks controlling the differentiation processes were monitored with a high throughput reverse phase protein microarray at 33 time points for 14 days to characterize adult adipose-derived stem cell differentiation [30]. Dynamic networks demonstrated key phosphoregulatory events in signal transduction pathways correlated with adipogenic differentiation. This is a novel way to understand the signaling architecture of mesenchymal stem cell differentiation and might be useful in developing network-targeted therapies for clinical applications. The dynamic protein phosphorylation networks, e.g. the insulin signaling network in adipocytes, were investigated by high-resolution mass spectrometry-based proteomics [31]. The integration of large-scale phosphoproteomics data predicted physiological substrates of several diverse insulin-regulated kinases, within which an Akt substrate, SIN1, a core component of the mTORC2 complex, was identified to regulate mTORC2 activity in response to growth factors, revealing topological insights into the Akt/mTOR signaling network. This particular study provides an excellent example to understand complex and dynamic signaling networks in tissues which contain numerous phosphorylation sites on proteins involved in diverse molecular functions.

## Focus on respiratory diseases

Network biomarkers and interaction networks were most studied in lung cancer among all kinds of respiratory diseases. Transcription factor profiling of lung adenocarcinomas in c-myc-transgenic mice suggested a model of a transcriptional regulatory network where different transcription factors acted in concert upon c-Myc overexpression [32]. Molecular networks for transcriptional regulation could explain partly the carcinogenic effect seen in mice with over-expression of the c-Myc oncogene. Similarly, the GATA2 transcriptional network was discovered to be requisite for RAS oncogene-driven non-small cell lung cancer [33]. Novel functional view of the crocidolite asbestos-treated A549 human lung epithelial transcriptome revealed an intricate network of pathways with opposing functions including cell death, cancer, cell cycle, cellular growth, proliferation, and gene expression [34]. Network Component Analysis and Pathway Crosstalk Analysis was performed to construct a regulatory network in human lung cancer (A549) cells which were treated with motexafin gadolinium (MGd), a metal cation-containing chemotherapeutic drug for 4, 12, and 24 hours [35]. This dynamic network of transcription and pathway crosstalk clearly demonstrated molecular mechanism of MGd-treated human lung cancer cells. After downloading the preprocessed microarray expression dataset from Gene Expression Omnibus database, our group [36] applied a new computational strategy for the identification and biological interpretation of new candidate genes in lung cancer and smoking by coupling a network-based approach with gene set enrichment analysis. Panels of top ranked gene candidates, major gene hubs and commonly involved pathways in both the smoking and cancer related network were identified. This new approach of bioinformatics for biomarker identification can probe into deep genetic relationships between cigarette smoking and lung cancer, although further validation in clinical settings is needed.

For diagnosis of lung cancer, a systems biology approach integrating microarray gene expression profiles and protein-protein interaction information was proposed to develop a network-based biomarker [37]. In addition, the network-based biomarker, acting as the screening test. About 40 significant proteins in lung carcinogenesis were identified on the basis of the network-based biomarker principle. In addition, the network-based biomarker acting as a screening test was shown to be effective to diagnose cancer in smokers with signs of lung cancer. Artificial neural network model built with the six serum tumor markers was shown to be able to distinguish lung cancer, from lung benign disease and normal people as well as from three common gastrointestinal cancers [38]. A transcriptome network analysis method was used to construct gene regulation networks on published microarray data and select candidate genes for squamous lung cancer [39]. The genes of SPI1, FLI1, FOS, ETS2, EGR1 and PPARG were defined as candidate biomarkers, although further validation is needed for clinical screening or diagnosis. A transcriptional network classifier containing 25-gene network signature for distinguishing adenocarcinoma from squamous cell carcinoma was inferred from the molecular profiles of 111 human lung carcinomas to characterize different subtypes of lung cancer [40]. Network-based approach was also used to predict the prognosis for lung cancer. The combination of physical and biological factors with a graphical Bayesian network framework was found to improve the overall prediction for local failure following radiotherapy in lung cancer [41]. A systems biology-based network approach using lung tissues for analysis was used to identify a cell cycle gene module and three hub genes as predictor of overall survival in lung adenocarcinoma patients [42].

A number of studies on the role of interaction networks and network biomarkers have been performed to explore molecular mechanisms and identify potential biomarkers associated with other respiratory diseases, though the specificity and repeatability remain unclear and further validation is needed. Network inference algorithms elucidated nuclear factor erythroid 2-related factor regulation of mouse lung oxidative stress and showed the promise for operating on high-throughput gene expression data to identify transcriptional regulatory and other signaling relationships [43]. Response network analysis of differential gene expression in human epithelial lung cells was used to compare the response to H5N1 infection with a more benign infection with Respiratory Syncytial Virus [44]. Characteristics of H5N1 infection compared to respiratory syncytial virus infection showed several immune response factors specific for each of these infections. Metabolic network in Pseudomonas aeruginosa-infected chronic cystic fibrosis lung demonstrated how the bacterial metabolism adapted over time and how the tradeoffs between growth and other important cellular processes shifted during disease progression [45]. A successful application of DNB on respiratory diseases was on a murine acute lung injury model driven by carbonyl chloride inhalation [46]. By applying DNB theory on the time-course microarray data from lung tissue RNA, a group of observable molecules were screened out at 8 hour, which formed a strong correlated subnetwork just before the occurrences of lung injury and thus provided a reliable early-warning signal [27]. Although this was an animal study with limited samples, it validated the effectiveness of DNB for the identification of toxicity mechanisms and the early diagnosis of carbonyl chloride induced acute lung injury.

## DNB with clinical bioinformatics

Clinical bioinformatics is an emerging science combining clinical informatics, bioinformatics, medical informatics, information technology, mathematics and omics science together [47]. There is an increasing need to emphasize clinical phenotypes and medical informatics in developing disease-specific DNB. Clinical bioinformatics has been suggested as a new way to integrate clinical symptoms, signs and measurements with human sample-generated bioinformatics for the development of disease-specific DNB [47]. It is challenging to adjust DNB to clinical symptoms and signs, disease development and progression, and therapeutic strategy. Clinical bioinformatics should be emphasized for DNB identification and validation to handle data preprocessing and consolidation, the data-driven search, verification, prioritization and biological interpretation of putative metabolic candidate biomarkers, as suggested by Baumgartner et al. [48]. It is also challenging to select proper data mining tools for analyzing clinical proteomic data, to design clinical studies like case–control or prospective cohort studies, or to translate new findings of disease-specific DNB to clinical application.

The importance of the integration of proteomic profiles and data with clinical bioinformatics was emphasized to bridge the gap between proteomics and diseases, e.g. acute and chronic organ injury, inflammation, and multiple organ dysfunction [49]. It is easy to understand that proteomics as a powerful tool help investigate the relationship between biological molecules and disease mechanisms, while difficult to integrate proteomics-based DNB with physiological and pathophysiological situations, with organ-, tissue-, type-, function-, disease-specific patterns, or with disease diagnosis, therapies and prognosis. A new protocol of disease-specific DNB identification and evaluation was developed by integrating proteomic profiles of inflammatory mediators with clinical informatics in patients with acute exacerbation of chronic obstructive pulmonary disease (AECOPD) [50,51]. Serum protein profiles from patients with AECOPD were assessed by different strategies of proteomics on days 1 and 3 of the admission day and the discharging day and correlated with clinical informatics by a Digital Evaluation Score System for assessing severity of patients. A panel of inflammatory mediators with dynamical changes during disease progression was demonstrated to be COPD specific or AECOPD specific biomarkers and correlated well with clinical bioinformatics. Such protocol can integrate proteomics with clinical informatics to explore a new way to validate and optimize disease-special DNB, which might be used clinically after further validation with a larger sample size.

## Conclusions

Disease-specific interaction networks, network biomarkers or DNB have the great impact in the understanding of mechanism interpretation, risk assessment, early diagnosis, illness monitoring, disease classification, stage and grade, effectiveness and toxicities prediction, as well as combinational therapies optimization. Protein-based DNB will provide more information to define the differences between the normal and pre-disease stages and point to early diagnosis. Network biomarkers, interaction networks and DNB have been applied in respiratory diseases and will face a number of opportunities and challenges. Clinical bioinformatics should be considered as a key approach to the identification and validation of disease-specific biomarkers.

## Review

The last few years have seen a fast development of network medicine, especially in cancer research. A number of interaction networks or network biomarkers have been proposed to be related to disease development, progression and therapeutic effects. Thus, they are of great potential for early diagnosis, prognosis prediction and efficacy prediction. However, there are also several challenges to translate the research of network based approach into clinical application. Firstly, since many findings were from in vitro cell line studies or current database, further validation studies in clinical settings will be of great importance. Secondly, network biomarkers, interaction networks or DNB are more complex than single biomarker and more difficult to develop, optional computational methods or algorithms are needed. Thirdly, the generation of network biomarkers or DNB usually need a three dimensional image of biomarker-biomarker interactions showing time-dependent stronger or weaker interactions among biomarkers in the network, which needs rich and integral data collection. All these make sense in respiratory diseases, in which area network based approach will also progress and have a bright future.

## Competing interests

The authors declare that they have no competing interests.

## Authors’ contributions

XD Wu contributed to collection of information and writing of the manuscript. LNC contributed to revision of the manuscript. XD Wang contributed to the critical revision of the manuscript. All authors read and approved the final manuscript.
